# Electrostatic reaction for the detection of circulating tumor cells as a potential diagnostic biomarker for metastasis in solid tumor

**DOI:** 10.7150/ntno.46928

**Published:** 2020-09-04

**Authors:** Zhiming Li, Xingping Liu, Weidong Zhang, Xuan Zhuang

**Affiliations:** 1 Institue of Reproductive Health, Tongji Medical College, Huazhong University of Science and Technology, Wuhan 430030, Hubei, China.; 2 Department of Urology, the First Affiliated Hospital of Xiamen University, Xiamen 361003, Fujian, China.; 3 Clinical Trial Management Platform, Jinhua Municipal Central Hospital, Jinhua 321000, Zhejiang, China.; 4 Department of Clinical Medicine, Fujian Medical University, Fuzhou 350005, Fujian Province, China.

**Keywords:** Circulating tumor cells (CTCs), Syngeneic tumor models, Nanoparticles, Bioelectricity

## Abstract

The detection of circulating tumor cells (CTCs) from blood samples is important to predict metastatic spread of cancer cells. However, effective quantification and identification of CTCs in solid tumors remain a challenge. Aerobic glycolysis is a hallmark of cancer cells, which makes cancer cells have more negative membrane potentials than that of normal cells. Herein, we reported a CTC isolation method with 80.7% capture efficiency based on electrostatic reaction, which was accomplished within 30 min in mimic clinical samples. Following *in vitro* verification using Lewis lung carcinoma (LLC1) (EpCAM-positive) and B16F10 (EpCAM-negative) melanoma cells, syngeneic tumor models were used to evaluate specificity and sensitivity of the surface charged nanoparticles. After subcutaneous implantation, blood was drawn from mice every three days, and CTCs were successfully detected in all implanted subjects. From 100 µl blood samples, the minimum amount found in blood was 9-34 CTCs on 3 day and the maximum was 94-107 CTCs on 15 day. Besides, the isolated CTCs cells remained viable and verified by re-implantation. This study confirms that our multifunctional nanoparticles are highly efficient in detecting CTCs in tumor metastasis and has huge potential in translational medicine.

## Introduction

Cancer is still a leading cause of death worldwide 1. The main cause of cancer patient death, however, is not the primary tumor but metastasis. It has been reported that circulating tumor cells (CTCs), tumor cells shed from solid tumor, at a daily rate of 3.2 × 10^6^ cells per gram of tissue, but over 50% CTCs quickly perish 2. What remains is about one tumor cell per 10^6^-10^7^ leukocytes 3. The rarity and significance of these tumor cells has led to the development of different technologies aimed to enrich for low population. At present, most of CTCs capture methods could be fall under two conceptual umbrellas: biomarker-based and biomarker-free 4. However, because of the complexity and variety of tumor antigen expression, there is no single biomarker which can achieve 100% error-free CTC capture. Fluorescence assisted cell sorting and *in situ* hybridization are highly specific, but often require exogenous labeling. Deterministic lateral displacement and centrifugal microfluidics are label-free tools, but cell physical differences may be too subtle, or cell perturbations may happen during centrifugal separation 5. Micropillars and graphene oxide nanomaterials have the special structure allows them to be easily combined with other biomolecules, but lack of a standard detachment method. It is well known that a great number of negative electrical charges are closely correlated with the secreted lactate acid of tumor cell surfaces 6. High levels of negative charge on tumor cell surfaces may become an outstanding feature, which could be isolated by electrical nanoparticles from the normal cells. Moreover, we hypothesized that it exists a close correlation between the number of CTCs and the time of tumor metastasis, which could be tested by cell-nanoparticle electrostatic interactions.

Bioelectricity usually means the flow of electrical currents, carried by mobile charged ions, across cell surface and along the exterior and interior ionic environment of cells 7. For example, bacteria could be isolated by the ion-exchange resins of electrostatic interaction chromatography (ESIC), through the electrostatic adsorption 8. We have developed an effective method for detecting *E. coli* at extremely low concentrations of 10 CFU/mL using surface-charged magnetic nanoparticles via electrostatic interaction 9. For cancer cells, it has been demonstrated that these metabolisms are fundamentally differ from normal cells, which have a much higher rate of glycolysis from “Warburg Effect” leading to a net negative charge on tumor cell surfaces 6. The phenomenon of “Warburg effect” can be commonly observed in cancer cells wherein cells ferment glucose to lactic acid via glycolysis even in the presence of oxygen. The physiological concentration of lactate, in blood and healthy tissues is about 1.5-3 mM 10, but in cancer tissues it can be present in up to 10-30 mM concentrations 11. Recently, we established an efficient method for isolating tumor cells from the ascitic tumor mice 12, while detection of CTC in solid tumor is still difficult. The challenge here is CTCs are relatively less in peripheral blood of solid tumor. It has been demonstrated that there was a significant difference in mobility between cells derived from the ascites and solid forms 13. Nevertheless, released sialic acids by the action of neuraminidase from the ascites and solid tumor were in common. Although lactic acid is not electrochemically active, the enzyme cofactor driving the reduction of pyruvate to lactate by lactic acid dehydrogenase, nicotinamide adenine dinucleotide (NADH), is oxidized to NAD+, which is electrochemically active at sufficiently negative potentials 14.

In this study, the electrically charged, fluorescent and superparamagnetic nanoparticles were developed for CTCs capture and metastasis monitor without using any antigens. Isolation of CTCs from mouse blood was investigated using two murine cancer cell line-derived tumor models, generated from mouse Lewis lung carcinoma (LLC1) and B16F10 cells that had previously reported to shed CTCs and metastasize to the lung 15. Through our strategy, we showed a successful isolation of CTCs with high capture efficiency. This study provides evidence that targeting negative surface charges of cancer cells offers a simple, effective method for monitoring cancer metastasis.

## Materials and Methods

### Nanomaterials

Iron (III) chloride hydrate (FeCl_3_·6H_2_O), hydrochloric acid (37 wt% aqueous solution), NH_4_OH (28 wt%), sodium acetate and ethylene glycol were purchased from Shanghai Chemical Reagent Company (Shanghai, China). Tetraethyl orthosilicate (TEOS), (3-aminopropyl) triethoxysilane (APTES) and fluorescein tetramethylrho-damine (TRITC) were obtained from Sigma-Aldrich (St. Louis, MO, USA). PEI (99%, Mw = 10000) was obtained from Alfa Aesar. Nanoparticles suspensions and all stock solutions were prepared using Milli-Q deionized water (18.2 MΩ•cm at 25 °C resistivity).

### Nanoparticle synthesis

The magnetic composite nanoparticles were firstly constructed by the solvothermal reaction. Briefly, 0.081 g of FeCl_3_·6H_2_O and 2.56g of sodium acetate were dissolved in 30 mL of ethylene glycol under magnetic stirring. 1.8 g urea and 0.3 g of polyacrylic acid (PAA) were added to above a mixture solution. After mixing and magnetic stirring for 30 min, the solution was transferred into a Teflon-lined stainless-steel autoclave and heated at 200 °C for 12 h. After the solution cooled to room temperature, the core-shell nanoparticles were formed and collected by a magnet. The product was washed by ethanol and deionized water three times.

To prepare the negatively charged nanoparticles (NP-), the core-shell nanoparticles was treated with 0.15 M HCl under sonication for 15 min and then were coated with silica through hydrolysis and polymerization of TEOS. To add the fluorescent group to prepared nanoparticles, APTES-TRITC complex was first reacted in ethanol under dark conditions overnight. TRITC fluorescent group was then grafted to core-shell nanoparticles via the reaction between APTES and hydroxyl groups on the surface of Fe_3_O_4_@SiO_2_. The product was washed by ethanol and deionized water three times. For the preparation of the positively charged nanoparticles (NP+), NP- were dispersed in PEI solution under gentle stirring and ultrasonic treatment for 3 h. The product was washed by ethanol and deionized water three times.

### Nanoparticles characterization

Structure and surface morphology of the nanoparticles were determined with a field emission transmission electron microscope (TEM) (JEM-2010). The zeta potential and particle size of nanoparticles were examined using a Malvern Zeta Sizer Nano series (Westborough, MA). Fluorescence was observed with a Carl Zeiss LSM5 EXITER laser scanning confocal microscope (Zeiss, Jena, Germany).

### Cell culture

LLC1 (mouse lung carcinoma) and B16F10 (mouse melanoma) cell lines were purchased from ATCC. Both cell lines were cultured in Dulbecco's modified Eagle's medium High-Glucose (DMEM, Gibco) supplemented with 10% foetal bovine serum (FBS, Gibco).

### Ethics statement

The mice were housed in the specific pathogen-free facility of Tongji Medical College, Huazhong University of Science and Technology. All animal experiments were carried out in accordance with the according to the Guide for the Care and Use of Laboratory Animal guidelines.

### Tumor growth and metastasis model

C57BL/6 and FVB mice used were 8-10 weeks old and obtained from Shanghai Institute of Medical Industry. LCC1 and B16F10 cell (1 × 10^6^ cells in 0.1 ml PBS) were injected subcutaneously into each mouse. Tumor growth were monitored from the time tumors became palpable until they reached 1000 mm^3^. Then tumor growth was recorded daily by vernier calipers measuring both the longitudinal (l) and the transverse (t) diameter. Tumor volume was estimated using the formula: tumor volume = (l × t^2^)/2.

### Blood collection

200 μl of blood samples were collected by retro orbital bleeding and transferred into K2-EDTA-coated tubes. To perform retro orbital blood withdrawal, mice were deeply anaesthetized with 1% isoflurane, and the microhematocrit is located in the medial canthus of the eye and directed caudally at a 30-45° angle from the plane of the nose.

### Cell immunostaining and enumeration

After nanoparticles isolation, cells were transferred to a glass slide and fixed with 4% paraformaldehyde for 5 min at room temperature. Then the cells were then blocked with 5% bovine serum albumin for 1 h at room temperature and incubated with specific primary antibodies: a PE-labeled anti-CD45 antibody, a FITC-labeled antiCK18 antibody (Abcam) overnight at 4 °C. After staining, the cells were imaged using an Axio Observer Z1 microscope (Zeiss) and manually enumerated.

### Cell viability and re-culture assays

Isolated CTCs were transferred from the tube to a cultivation plate using FBS-enriched DMEM medium (10%), under normal cancer cell incubation environments (37 °C, 5% CO2).

### Histopathological examination

After sacrifice of mice, a portion of tumor tissues were dissected and fixed in 4% paraformaldehyde overnight at 4 °C. Fixed tumor tissues were routinely processed and embedded in paraffin, then serially sectioned at 5 μm and stained with hematoxilyn and eosin (H&E). For immunochemistry staining, tissue sections were treated with 0.01 M sodium citrate (pH 6.0) at 95 °C for 30 min, followed by inactivation of endogenous peroxidase with 3% hydrogen peroxide, blocking by 5% bovine serum albumin. Sections were incubated overnight at 4 °C with primary antibodies against programmed death-ligand 1 (PD-L1) antibody (Abcam). Staining was performed with the UltraView Universal DAB Detection Kit (Ventana) according to the manufacturer's recommendations.

## Results

### Characterization of NP+

**Figure 1:**
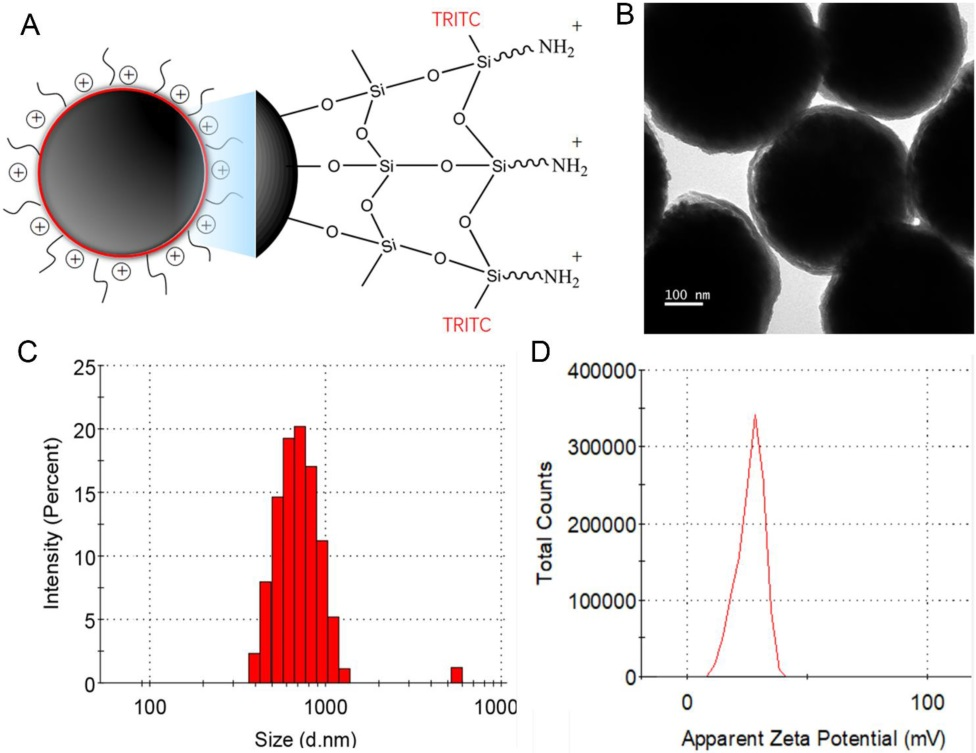
** Design and characterization of the nanoparticles**. (A) Schematic diagram of the multi-functional nanoparticles with electronical charge and fluorescent signal. (B) Transmission electronic microscopy image of the nanoparticles showing a thin layer of silica coating. (C) Dynamic light scattering analysis showing size and distribution of the nanoparticles; (D) Zeta potential distributions of the nanoparticles.

A schematic diagram of the electrically charged, fluorescent, and superparamagnetic nanoparticles is showed in Figure 1A. Superparamagnetic Fe_3_O_4_ nanocores were designed for fast separation of the attached cells by a magnet. TRITC is a bright orange-fluorescent dye, which could be utilized for direct imaging of NP+ bound on the CTCs. PEI molecules with many amine groups are bound to the SiO_2_-OH groups via electrostatic interaction, which provides high density of positive charge sites on surface of nanoparticles. TEM result displayed a uniform silica coating (30 nm) on the nanoparticle surface with an average particle diameter of 400 nm (Figure 1B). After surface functionalization, dynamic light scattering (DLS) displayed a narrow size distribution (the polydispersity index is 0.336) with an increased average diameter of 600 nm (Figure 1C). The presence of some bigger nanoparticles may cause an increase light scattering, shifting the measured particles size towards larger values 16. Most of NP+ exhibited a peak zeta potential of + 28.1 mV in deionized water (pH 7.0), which were showed in Figure 1D. According to the obtained results, the surface-charged nanoparticles dispersed well in aqueous solution under the neutral condition and could be used into CTCs capture.

### Capability of NP+ based cell capture

**Figure 2:**
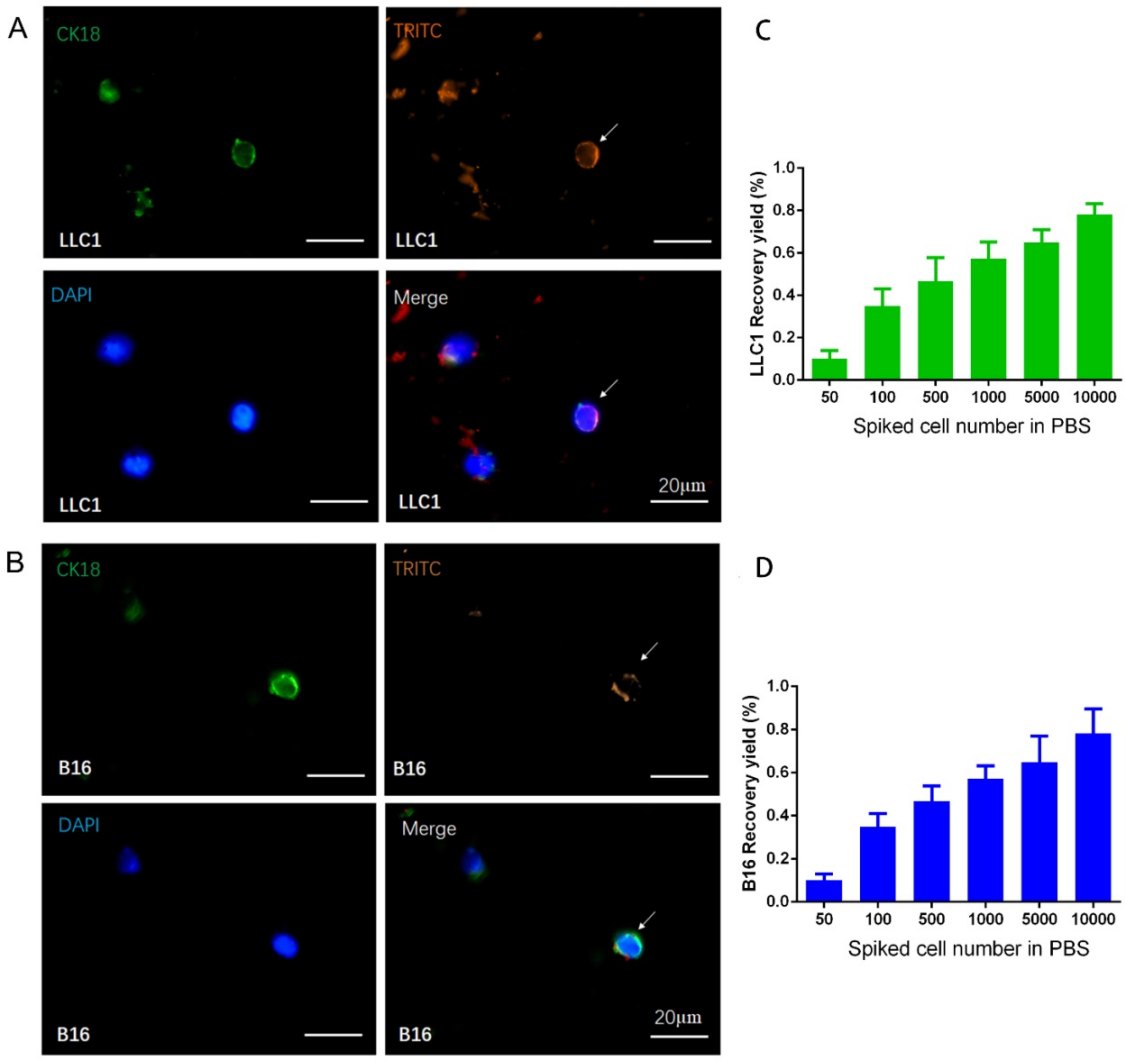
** Representative fluorescent images and recovery yields of cancer cells isolated from PBS.** Captured LLC1 (A, C) and B16F10 (B, D) murine cancer cells were fixed and stained for CK18 (green). Nuclei were highlighted with DAPI (blue). TRITC (orange) was used to label the nanoparticles. Recovery yield of NP+ at series concentrations of spiked cancer cell (50, 100, 500, 1000, 5000, 10000 cells / mL) in PBS solution.

**Figure 3:**
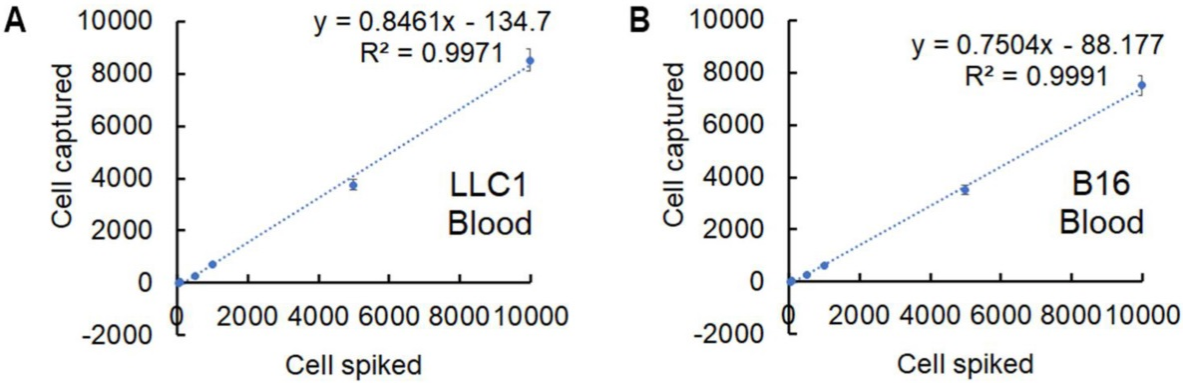
** Capture efficiency of the cancer cells spiked in the whole blood.** Capture efficiency of NP+ at series concentrations of spiked LLC1 (A) and B16F10 (B) cells in the blood samples. Data represent the mean ± standard deviation (SD) of three independent experiments, each performed in triplicate.

To further examine the potential of the designed nanoparticles for the isolation and detection of CTC in animal models, the capture effect corresponding to LLC1 and B16F10 cancer cells incubated with NP+ in PBS solution were first tested. As displayed in Figure 2A and 2B, fluorescence images revealed that captured cells were positive for CK18 (green), TRITC (orange) and DAPI (blue), indicating that the spiked cancer cells could be effectively “pulled out” from the tube by a magnet upon the electrostatic interaction. The fluorescence analysis illustrated that the nanoparticles are all in spherical shape around the cells, having an orange color from TRITC. Furthermore, the recovery rate of LLC1 and B16F10 cancer cells was displayed in Figure 2C and 2D. Different numbers of LLC1 and B16F10 cells (50, 100, 500, 1000, 5000, and 10000) spiked in 1 mL PBS solution were respectively incubated with NP+ (40μg). After magnetic separation, the samples were analyzed with the immunofluorescence. It can be seen that the increased recovery yield is associated with an increased cell number. About 65.6% cancer cells were captured at 500 spiked cells. The recovery yield is nearly 82.8% when higher number of cells (5000 and 10000) in 1ml PBS solution. Even in low number cell groups of 50 and 100, the recovery yield is up to 17.6% and 35.2%, respectively. Capture efficiency is comparatively lower probably due to loss of cells in the procedures of immunofluorescent staining, such as such as fixation, blocking and wash. Next, we investigated the capture efficiency via series of parallel spiked experiments using the whole blood samples. We counted the number of enriched cells through biomarkers staining and found that capture efficiency up to 80.7% under 10000 spiked cells (Figure 3). Compared with the recovery rate in PBS solution, the value is slightly lower in blood that contains many different nonhematologic populations. We observed similar results dealing with B16F10 cancer cells, indicating a good reproducibility of the approach. In addition, the captured purity of cells isolated by NP+ from blood samples was found to be over 85% in a mixture of 10000 LLC1/B16F10 cells and 1 mL whole blood (as shown in Figure S1A). The very low population of captured background cells from an initially extremely high number of leukocytes demonstrated the low binding rate of NP+ to leukocytes (as shown in Figure S1B).

### Characterization of tumor growth and CTCs in the animal models

**Figure 4:**
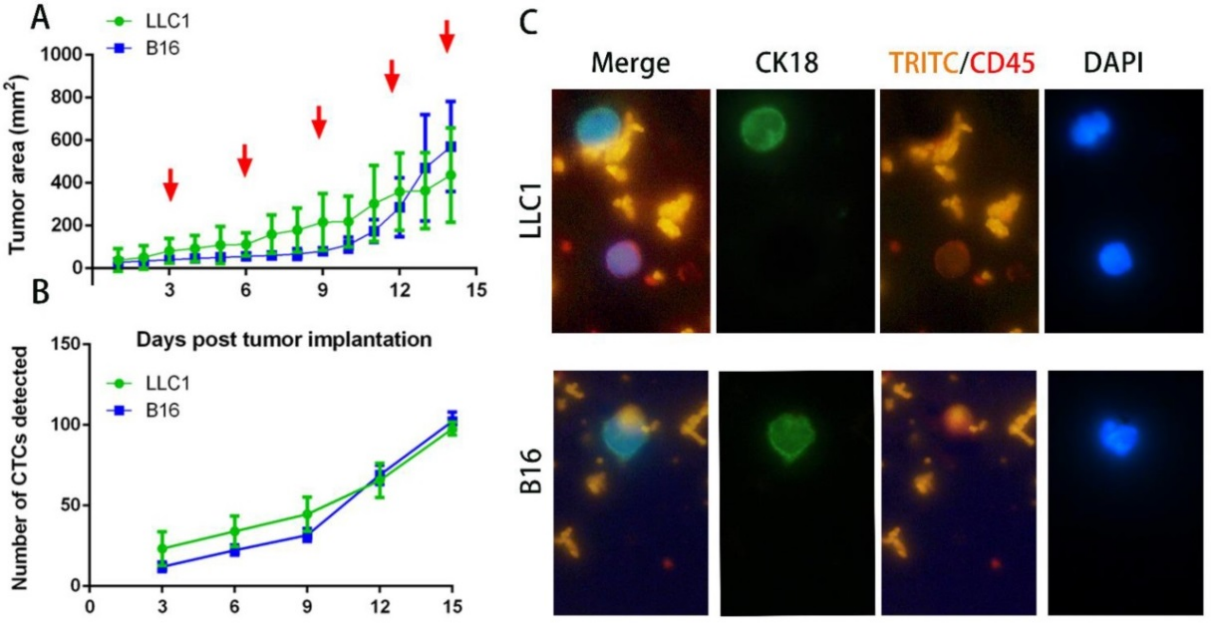
** Tumor growth curves, recovered numbers, and representative fluorescent images of CTCs.** (A) Tumor growth curves show an increased trend after post tumor implantations of LLC1 and B16F10 cells. Blood was drawn on days 3, 6, 9, 12, and 15 days (red arrow). Data represent the mean ± SD of three independent experiments, each performed in triplicate. (B) Number of CTCs isolated by NP+ from blood samples at above days. (C) Representative fluorescent image of CTCs isolated from individual syngeneic models. Nuclear staining (blue), CK18 (green), TRITC (orange), CD45 (red).

After implantation of 2 × 10^6^ cancer cells into C57BL mice subcutaneously, individual tumor volume was measured and plotted as a tumor growth time, and blood was collected for CTCs isolation. Tumor growth was calculated every day, and blood was drawn on days 3, 6, 9, 12 and 15 post implantations. Figure 4A shows the tumor growth curves of LLC1 and B16F10 implanted mice. It can be seen that there was similar growth trend for two murine cancer cell lines, as expected. Figure 4B illustrated that the number of isolated CTCs from blood samples is increased with tumor progression until reaching the maximum level at day 15 for all individuals. Notably, the minimum amount identified in blood was 9-34 CTCs on 3 day, and the maximum was 94-107 CTCs on day 15. All mice were euthanized at day 16, and lungs, tumor and blood were collected for further analysis. It has been demonstrated that blood collected from the retroorbital sinus of tumor models had high numbers of epithelial cells (CK+/DAPI+/CD45-), suggesting that this blood collection strategy is probably suitable for CTC identification in cancer metastasis. The cells with cytokeratin- and DAPI-positive staining were identified as CTCs, whereas the CD45-positive staining and nucleated cells were considered as leukocytes. In our immunofluorescent analysis, NP+ were bound on tumor cells but not attached on the surface of CD45-positive cells (Figure 4C). In addition, we also cultured the recovered CTCs to explore the cell viability after magnetic separation. The cells did not show detectable changes in visual morphology underwent three passages.

**Figure 5:**
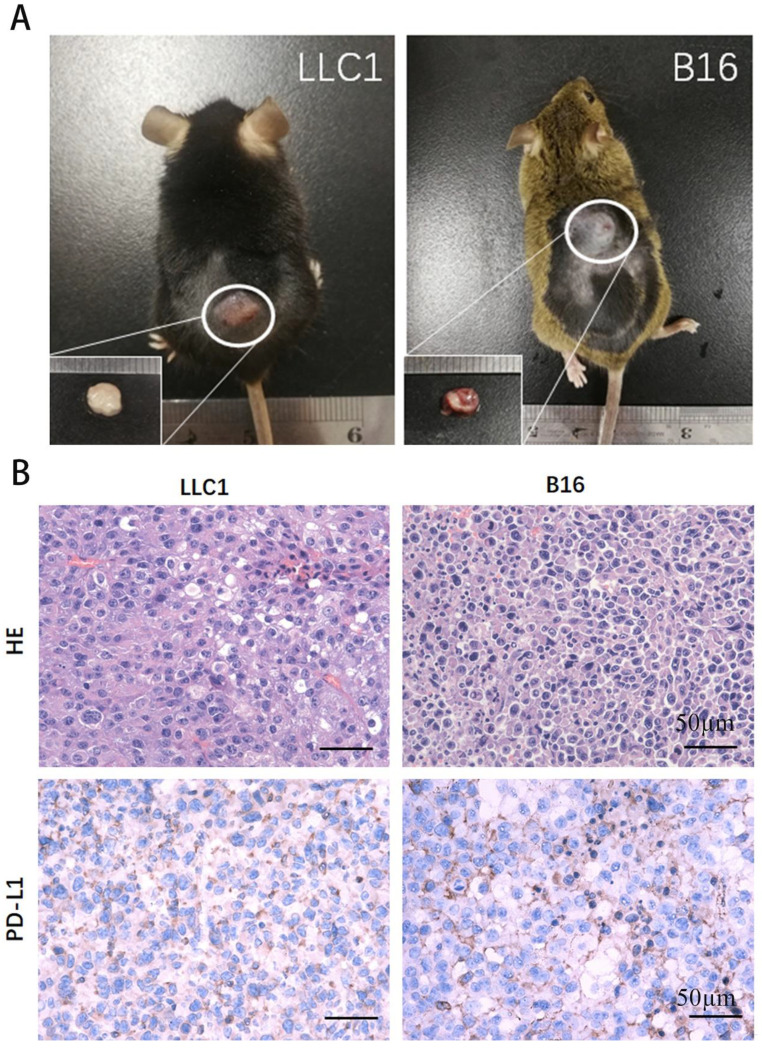
** Viability analyses of the recovered LLC1 and B16F10 cells.** (A) C57BL and FVB mice were implanted subcutaneously with recovered LLC1 and B16F10 cells. The subcutaneous tumor was excised at 16 day. (B) The histological sections were stained with H&E and with antibody PD-L1. Scale bars, 50 mm.

**Figure 6:**
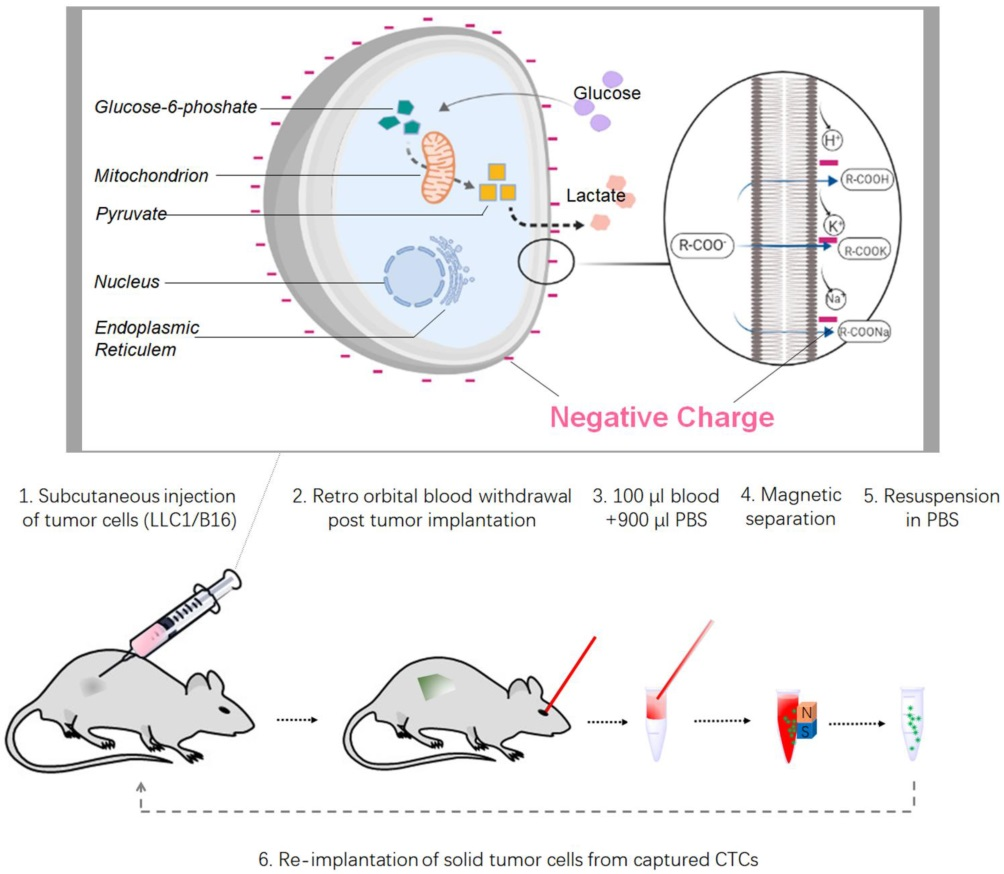
** Schematic diagram of CTCs capture from solid tumor through electrostatic reaction.** The cell surface of lactate is an end product of the glycolysis of glucose. The loss of highly mobile lactate from cancer cell cytoplasm will result in the remove of labile inorganic cations (Na+ and H+) to form lactate salts and acids. When cancer cells continuously remove cations from the cytoplasm due to the persistent activation of aerobic glycolysis, generation of net negative charges on cancer cell surfaces becomes inevitable. Based on these findings, we designed a workflow of CTC capture platforms and then validated by syngeneic models using two murine cancer cell lines.

It is widely accepted that CTCs are contributory precursors for cancer metastasis, such as lung cancer and melanoma. We implanted recovered CTCs into both C57BL and FVB mouse. At 16 days, the mice were sacrificed and subcutaneous tumors were harvested for visual and histopathological analysis (Figure 5A). The results showed that recovered CTCs caused tumor growth in animals. It is indicated that the positively charged nanoparticles-based CTCs capture has little influence on viability and proliferation of isolated cells, which is benefit for biological study subsequently. In particular, the captured CTCs could be cultured and used for drug-resistant test. Additionally, subcutaneous tumors from LLC1 and B16F10 CTCs implanted animals were sectioned and analyzed by H&E and immunohistochemistry (Figure 5B). It has been demonstrated that tumors can escape immune recognition and attack by upregulating PD-L1, a well-characterized immune checkpoint 17. In both tumor types, PD-L1 positive cells were remarkably observed by immunohistochemistry (Figure 5B). Taken together, we summarizes the workflow for the systematic development of CTC detection platforms in this study (Figure 6): (1) Solid tumor models established by dorsal subcutaneously LLC1/ B16F10 cancer cells injection, (2) Blood collection from the retroorbital sinus of implanted tumor animals, (3) Resuspend 100 μl blood samples in 900 μl PBS for CTCs capture, (4) The NP+ bound-cancer cells attached to the side of the tube by a permanent magnet, (5) Resuspend the isolated CTCs in PBS and enumerated via staining, (6) Implanted tumor model generated by cultured CTCs cells.

## Discussion

The most profound hallmark of cancer is the high rate of glycolysis, which was widely studied in cultured cancer cells but also extensively employed in cancer clinical settings (such as PET imaging) 18. Rapidly growing tumor cells rely on high levels of glucose uptake and lactate secretion, which are two most distinguishable metabolic behaviors. Therefore, high levels of negative charge on cancer cell surfaces could be used as a distinguishing characteristic and then isolated by electrical nanoparticles from the normal cells. Here, we found that the electrostatic reaction-based sorting strategy via NP+ indeed offer a good opportunity to capture heterogeneous cancer cells without using the specific antigens. Electrical charged nanoparticles avoid the immobilization of antigen on magnetic beads, which overcome the limitation caused by lacking of a standard attachment method. This isolation strategy is promising for rare and highly heterogeneous cancer cell isolation. Moreover, the viability of isolated CTCs is not to be influenced by electronical charged nanoparticles, which is more important in the further studies.

Over the past decades, CellSearch system remains the most common method of CTC enumeration. CellSearch system detected EpCAM expressing CTCs, which were identified as pan-cytokeratin (panCK)+/CD45-/DAPI+ cells. panCK (CK8/CK18/CK19) are the markers of epithelial cells, and CD45 negative expressing represents which are not blood cells 19. It has been reported that CK18 is a marker for both lung adenocarcinoma 20 and melanoma 21. EpCAM has a high expression in nearly all epithelial tumor types, while tumors of mesenchymal lineage such as melanomas and sarcomas have no expression 22. Our electrostatic reaction-based approach is able to effectively capture EpCAM-negative CTCs. Epithelial to mesenchymal transition (EMT) is the process whereby epithelial cells are transformed into mesenchymal cells, which is a route for cancer cells from a primary tumor to metastasis. An interesting external determinant of EMT is the acidic microenvironment, which is a consequence of tumor cell specific metabolism: aerobic glycolysis. Mesenchymal cancer cells exhibit higher aerobic glycolysis than that of epithelial cancer cells 23. Thus, it seems that that metabolic pattern is more homogenous than the EpCAM expression of CTCs. The unique pattern of cancer metabolic mechanism provides an important clue for *in vitro* drug sensitivity and resistance test using the enriched CTCs.

It has been known that cancer cell surface molecule expression has systematic inconsistence between *in vitro* and *in vivo* from the transcriptomic data 24. Therefore, various mouse models have been developed in CTCs capture studies, including syngeneic models (4T1 murine breast cancer cell line) 25, transgenic models (overexpressing EGFR) 26, and patient-derived xenografts (PDX, triple negative breast cancer patient-derived xenograft) 27, etc. In our study, we established a syngeneic model using murine cancer cells LLC1 and B16F10, which was chosen due to the ability to quickly metastasize to the lungs 15, 28. *In vivo* metastasis models are increasingly being used to study the correlation between the count of CTCs and tumor progression. With the development of next generation sequencing technologies, we can easily analyze the genomic and transcriptomic profiles of single CTCs which provided new insights into the molecular landscape of cancers 29. Recent finding highlighted the potential clinical significance of CTC molecular characterization as a valuable material to support precision medicine 30. We will then expect future studies in which CTCs may play more roles in monitoring cancer patients under the treatment of immunotherapy regimens. However, as PDX tumor models are produced in immunodeficient mice they could not provide a panacea for immunotherapy testing, and, so far, preclinical assessment of cancer immunotherapy depends on syngeneic models. Once we confirmed that our strategy can be employed into identify EpCAM positive and EpCAM negative expressed cancer cells, we then moved on to establish a syngeneic model using two cancer cells, for *in vivo* validation and metastasis monitoring. CTCs were successfully detected from the collected blood samples during the metastasis of tumor, indicating that this method may serve as an auxiliary diagnostic tool for monitoring tumor dynamics in the clinic.

## Conclusion

This study provided an effective and practicable CTCs capture strategy based on the high rate of glycolysis in cancers. Through this method, CTCs can be fast captured within 30 min from the blood samples by an external magnetic field. The heterogeneous CTCs were successfully isolated regardless of their cell surface biomarker expressions through electrostatic interaction. Notably, our findings give a different perspective on the fundamental understanding the bioelectricity of cancer cells, which may pave a new path for developing the approaches in cancer diagnosis and therapeutics.

## Supplementary Material

Supplementary figure S1.

## Abbreviations

CTCs: Circulating tumor cells
NP: Nanoparticles
EpCAM: Epithelial cell adhesion molecule
DLS: Dynamic light scattering
DAPI: 4′,6-diamidino-2-phenylindole
TRITC: Tetramethylrhodamine
PAA: Polyacrylic acid
TEOS: Tetraethyl orthosilicate
TEM: Transmission electron microscopy
NP+: Positively-charged nanoparticles
LLC1: Lewis lung carcinoma
PD-L1: Programmed death-ligand 1
H&E: Hematoxilyn and eosin
PET: Positron emission tomography
SD: standard deviation
