# Supplementary material for: Electrostatic reaction for the detection of circulating tumor cells as a potential diagnostic biomarker for metastasis in solid tumor
Source: Nanotheranostics. 2020 Sep 4;4(4):233–41. doi: 10.7150/ntno.46928 (PMC7484632; doi:10.7150/ntno.46928)
Supplement: Supplementary file 1 — Supplementary figure S1. [file ntnov04p0233s1.pdf]

Electrostatic reaction for the detection of circulating tumor cells as a potential diagnostic biomarker for metastasis in solid tumor

Zhiming Li[1,2*], Xingping Liu[1], Weidong Zhang[3], Xuan Zhuang[2,4*]

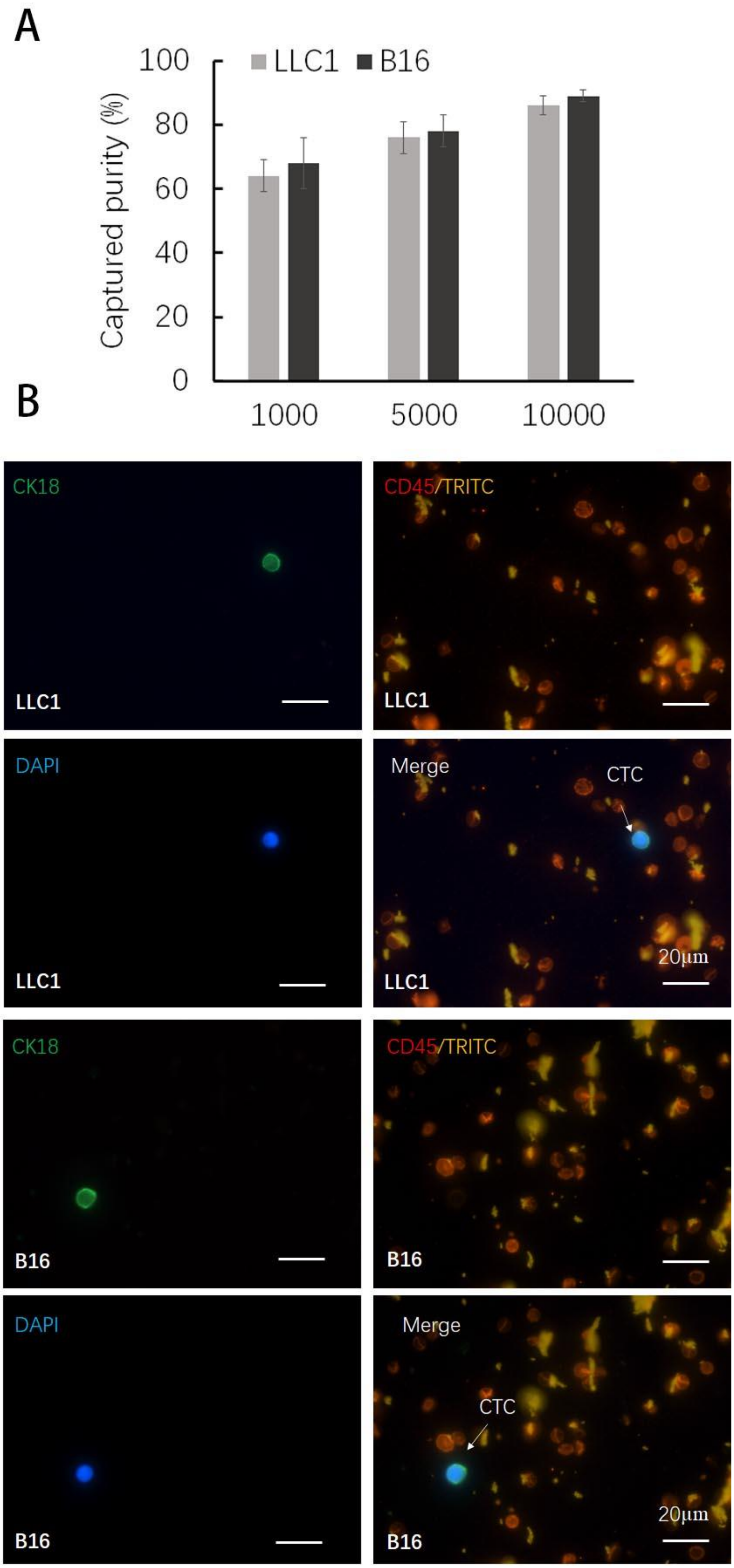
A
100
80
60
40
20
0
Captured purity (%)
LLC1
B16
1000
5000
10000
B
CK18
LLC1
CD45/TRITC
LLC1
DAPI
LLC1
Merge
CTC
20μm
LLC1
CK18
B16
CD45/TRITC
B16
DAPI
B16
Merge
CTC
20μm
B16

Figure S1. The purity and representative image of CTCs captured from the whole blood samples. (A) Capture purity (ratio of captured cancer cells to total captured cells) of cells mixed with 1 mL whole blood. (B) Fluorescent images of cancer cells isolated from the whole blood samples. Captured cells were then fixed, blocked, and stained for CK18 and CD45. Nuclei were highlighted with DAPI. TRITC was used to label the nanoparticles. The scale bar is 20 μm.
